# Clinical, Radiographic, and Patient-Perceived Outcome After Radial Hemi-Wrist Arthroplasty With a New Implant: 20 Cases With 5-Year Follow-up

**DOI:** 10.1177/15589447231151427

**Published:** 2023-02-08

**Authors:** Daniel Reiser, Marcus Sagerfors, Per Wretenberg, Kurt Pettersson, Per Fischer

**Affiliations:** 1 Department of Orthopaedic and Hand Surgery, Faculty of Medicine and Health, Örebro University, Sweden; 2 Karlskoga Hospital, Sweden

**Keywords:** hemi-wrist arthroplasty, arthritis, osteoarthritis

## Abstract

**Background:**

Distal component loosening is a common mode of failure in total wrist arthroplasty (TWA). A radial hemi-wrist arthroplasty (RHWA) has the potential to avoid problems related to the distal component in TWA. The aim of this study is to investigate clinical outcomes following surgical treatment with a new RHWA design.

**Methods:**

In this pilot study of 20 consecutive RHWAs, patients were assessed preoperatively and postoperatively for range of motion, grip strength, Visual Analog Scale (VAS) pain scores, and functional scoring using Patient-Rated Wrist Evaluation (PRWE), Disabilities of the Arm, Shoulder, and Hand (DASH), and Canadian Occupational Performance Measure. Radiographs were analyzed at 12 months and 5 years (mean, 5.1 years) postoperatively.

**Results:**

A total of 46 secondary surgeries were undertaken in 16 wrists, including 7 revisions. Another 6 patients are waiting for revision to radiocarpal arthrodesis. In non-revised patients, the DASH and PRWE scores improved, and wrist range of motion remained largely unchanged except for wrist flexion, which decreased. The VAS pain score during activity was reduced, and hand grip strength remained largely unchanged.

**Conclusions:**

The new implant resulted in improved functional scoring and improved VAS pain scores in non-revised patients, but many cases needed secondary surgery due to persistent pain. The high revision rate is a major concern, and further use of the implant in its current form cannot be recommended.

## Introduction

Painful wrist arthritis is often divided into noninflammatory and inflammatory causes.^1-3^ When involving the lunate fossa, surgical treatment options are usually restricted to radioscapholunate arthrodesis, radiocarpal arthrodesis (RCA), or total wrist arthroplasty (TWA),^4^ but all these procedures have limitations. Radioscapholunate arthrodesis can preserve some wrist motion, but nonunion rates of up to 21% and progressive arthritis of the midcarpal joint may require additional surgery.^5-7^ Total wrist arthroplasty is a motion-preserving procedure, but although implant designs have evolved over the past decades, implant failure due to distal component loosening remains non-negligible.^8-10^

Problems related to distal component loosening in TWA could be obviated with a hemi-arthroplasty design, but the results so far have been somewhat disappointing.^11,12^ Polyethylene against bone articulation was associated with high rates of synovitis secondary to polyethylene wear, and metal against bone articulation was associated with high rates of bone erosion.^11,12^ Carbon-fiber-reinforced poly-ether-ether-ketone (CRF-PEEK) is a composite material with an elastic modulus close to cortical bone.^13^ A new modular radial hemi-wrist arthroplasty (RHWA) device has been proposed using an articulation of CFR-PEEK, potentially reducing wear on both the implant side and articulating bone. In a previous study, the new RHWA was tested in a cadaveric model with favorable results.^14^ The modularity of the articular component allows for potential revision to a TWA without removal of the radial stem. The aim of this pilot study was to assess patient-related outcome measures in 20 patients operated on with the new RHWA design and to determine the possible need for improvement in implant design and instrumentation.

## Method

The study was registered in the Swedish Public Trials Registry *FoU i Sverige* (registration number 174601). The study was undertaken at the University Hospital in Örebro, a tertiary referral center in Sweden. Twenty consecutive wrists were included. Informed consent was obtained from all individual participants included in the study. Several surgical options are available for wrist arthritis. In cases with radius-scaphoid arthritis and preservation of the capitate-lunate articulation, a proximal row carpectomy (PRC) is a well-established option.^4^ In this study, indications for surgery were inflammatory or noninflammatory wrist arthritis involving the lunate fossa, with unsustainable pain after a period of nonoperative treatment procedures. The involvement of the lunate fossa excluded PRC as a surgical treatment option.^4^ The decision for surgery was made in consultation with the patient after assessing the amount of pain and the need to preserve motion in the wrist.

**Figure 1.**
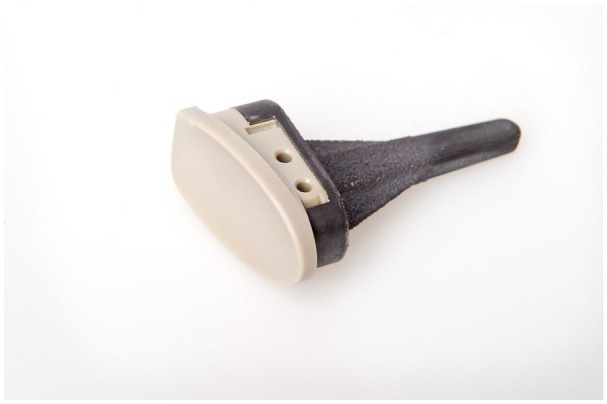
The new radial hemi-wrist arthroplasty design.

**Figure 2.**
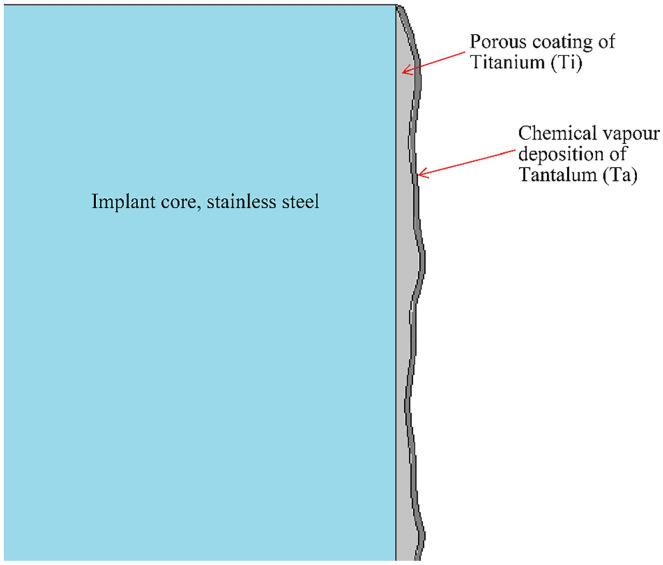
Framework of the radial stem in radial hemi-wrist arthroplasty.

The implant used was a new RHWA design manufactured by Trimed Inc. (Figure 1) and developed in cooperation with one of the authors (K.P.). The articulation is made of CRF-PEEK with an elastic modulus similar to that of the cortical bone.^13^ The radial stem has a press-fit design with a central core of stainless steel, a porous coating of titanium (Ti), and a thin outer layer of tantalum (Figure 2). Tantalum has higher porosity than Ti, favoring bone ingrowth and fixation strength,^15,16^ and has been used in the construction of acetabular cups used during revision of failed total hip arthroplasties with excellent results.^17^ The radial component has wing-like expansions, intended to increase rotational stability. The CRF-PEEK liner is modular and attached to the radial component with a snap-fit mechanism, making conversion of the RHWA to a TWA possible without exchange of the radial component. The CRF-PEEK liner comes in 2 different component sizes, designed for the options of preservation or removal of the proximal carpal row. The distal part of the radial stem, holding the CRF PEEK liner, has a flat vertical surface that rests directly on the cortical bone, potentially reducing the risk of stress-shielding.^18^

### Surgical Technique

All primary operations were performed by 1 surgeon (K.P.). The radiocarpal joint was approached through a dorsal straight longitudinal incision in line with the third metacarpal. After incising the fourth extensor compartment in a Z-shaped manner, the dorsal surface of the distal radius was exposed subperiosteally. The radiocarpal joint capsule was opened via a longitudinal T-incision, with care taken to preserve the lunotriquetral and scapholunate ligaments. The distal radius was resected straight horizontally, sparing the distal radioulnar joint. Positioning of the radial component was assessed using perioperative radiography. The articular surface was attached to the radial component by a snap-fit mechanism, and the stem was inserted into the radius. After reducing the carpus to the articular surface, passive movement of the radiocarpal joint was tested for the assessment of possible impingement. If conflict existed between the lunate and the ulnar side of the modular CRF-PEEK liner surface, the lunate was resected. The capsule was closed using absorbable sutures. Postoperatively, the wrist was immobilized in a cast for 2 weeks, after which active mobilization was begun under the guidance of a hand physiotherapist. Six weeks postoperatively, the patient was able to use the wrist fully, with no load restrictions.

### Clinical Evaluation

The following outcome measurements were registered by a hand therapist at our department prior to the operation and 1, 2, and 5 years postoperatively: range of motion (ROM) (flexion, extension, radial deviation, ulnar deviation, pronation, and supination); pain at rest and during activity using the Visual Analog Scale (VAS); Disabilities of the Arm, Shoulder, and Hand (DASH) score; Patient-Rated Wrist Evaluation (PRWE) score; Canadian Occupational Performance Measure (COPM) performance and satisfaction score; hand grip strength (kg); key pinch strength (kg); and tip pinch strength (kg). The Swedish validated versions of PRWE, DASH, and COPM were used.^19-21^

The PRWE is a 15-item questionnaire rating pain and disability equally. The maximum score is 100, and a higher score indicates more pain and functional disability. The DASH consists of a 30-item questionnaire evaluating the function of the whole upper extremity, covering both pain and disability. The maximum score is 100, with 0 indicating no disability and 100 the severest disability. The COPM includes 2 variables: COPM satisfaction and COPM performance. Through a semi-structured interview, the patient is asked to define 5 problems in daily living, rate their own level of performance, and rate their satisfaction with the performance of each of the 5 identified problems. The scoring ranges from 0 to 10, where 0 indicates very poor performance and low satisfaction. A physiotherapist using a goniometer recorded the ROM. Pain was evaluated both at rest and during activity, according to the VAS (in a range from 0 to 10, where 10 represents the worst pain imaginable). The mean of 3 attempts was recorded for tip pinch and key pinch strength using a pinch gauge (North Coast Medical Inc., Gilroy, California) and for hand grip strength using a hydraulic hand dynamometer (North Coast Medical Inc.).

### Radiographic Assessment

Anteroposterior examination and lateral radiographic examination were performed preoperatively, postoperatively, and at 1 and 5 years postoperatively. Radiographs were assessed for the presence of radiolucency around the radial stem and for subsidence or tilting of the implant. Ulnar carpal drift was measured as the difference between a vertical line in the center of the radius and a vertical line in the center of the capitate. When comparing the preoperative and postoperative radiographs, an increase in the ulnar carpal drift of ≥ 2 mm was considered to be a true ulnar carpal drift.

### Statistics

Patients who underwent revision surgery were considered to be failures and were not included in the statistical analysis of the clinical measurements. Here, revision surgery is defined as an exchange of the whole or parts of the prosthesis, or removal of the implant. As the data were non-normally distributed (Shapiro-Wilk test, data not shown), the difference between preoperative values and postoperative values was assessed using the Wilcoxon sign-rank test. Due to the low number of cases available for the 5-year follow-up, statistical assessment comparing the 5-year outcome data with the preoperative data was not undertaken. A corrected *P* < .05 was considered to be statistically significant.

## Results

The mean age at the time of primary RHWA was 58 years. The study included 20 wrists in 18 patients; 2 patients had bilateral surgery on the indication of rheumatoid arthritis (RA). The group of inflammatory wrist arthritis (n = 10) included 8 cases with RA, 1 with psoriasis arthritis, and 1 with pelvospondylitis. The non-inflammatory wrist arthritis group (n = 10) included 2 cases with Kienböck disease, 4 with scapholunate advanced collapse wrists, 2 with primary wrist osteoarthritis, and 2 with secondary wrist osteoarthritis after distal radius fracture (Table 1).

**Table 1.** Baseline Data.

Male/female	Mean age (range)	IWA/non-IWA	Side right/left
7/11	58 (35-76)	10/10	9/11

*Note.* Two patients underwent bilateral surgery. IWA = inflammatory wrist arthritis; non-IWA = non–inflammatory wrist arthritis.

All primary surgeries were performed by the same surgeon (K.P.) using the smaller component size of the CRF-PEEK liner, with preservation of the whole or parts of the proximal carpal row. During the study period, 1 patient died from a disease unrelated to the procedure; thus, 19 wrists were available for follow-up 5 years postoperatively. The patient who died underwent revision surgery with an exchange of the CRF-PEEK liner at 12 months after primary surgery.

The mean follow-up time was 4.8 years (range: 4-5.8 years). At the latest follow-up, 7 of the 19 wrists had been converted to an RCA and 1 to a TWA. The revised cases were not included in the statistical analysis, but charts were assessed to verify the number of reoperations. Of the 11 remaining cases, 6 are waiting for revision surgery, with planned removal of the implant and conversion to an RCA. Only 5 patients completed the latest clinical follow-up at the clinic. The rest were contacted through phone by a hand surgeon who had not participated in the initial surgery.

**Figure 3.**
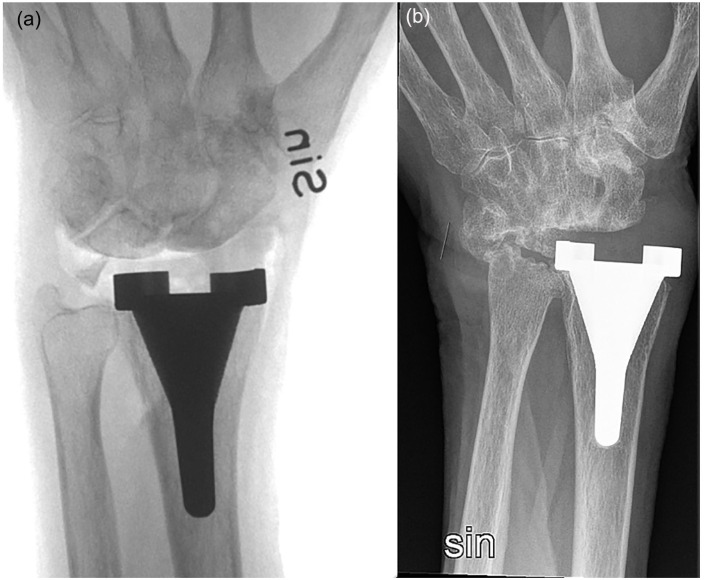
Anteroposterior radiographs (a) preoperatively and (b) 1 year postoperatively.

**Figure 4.**
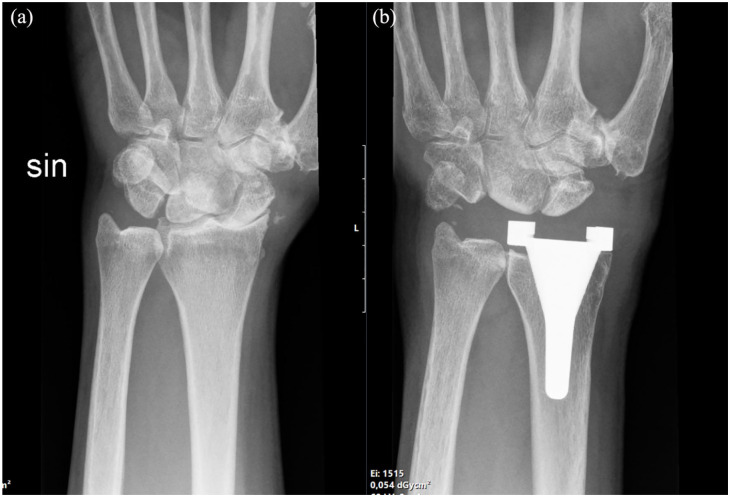
Anteroposterior radiographs (a) preoperatively and (b) 1 year postoperatively.

Radiographic assessment showed signs of radiolucency around the radial stem in 1 case, 1 year postoperatively. The patient did not undergo revision surgery because of no pain, and radiolucencies remained unchanged at subsequent follow-ups. However, bone erosions were noted on the articulating bones on the carpal side in 15 of 20 wrists (Figure 3). Ulnar carpal drift of ≥2 mm and carpal rotation were noted in 15 of the 20 wrists 1 year postoperatively, upon a comparison of the preoperative radiographs and postoperative radiographs (Figures 3 and 4). No signs of radiolucency were found around the implant in patients completing the 5-year follow-up.

Wrist flexion was significantly reduced at the 1-year follow-up but not at the 2-year follow-up (Table 2). No statistically significant difference was found when comparing the preoperative extension, radial and ulnar abduction, supination, pronation, and strength measurements with the postoperative values. The patients reported reduced pain at rest and during activity, ranging from median VAS 8.0 to 5.0 after 2 and 5 years postoperatively. Although COPM satisfaction improved significantly after 2 years, COPM performance remained unchanged. The DASH and PRWE scores improved significantly after 2 years compared with the preoperative values (Table 2).

**Table 2.** Clinical Evaluation.

Variable	Preoperative (n = 20)	1 year postoperatively (n = 20)	2 years postoperatively (n = 11)	5 years postoperatively (n = 5)
Wrist flexion	45° (30°-55°)	30° (20° to 40°)**	35° (20° to 50°)	25° (13°-60°)
Wrist extension	25° (20°-45°)	25° (16° to 38°)	25° (15° to 50°)	35° (8°-63°)
Radial abduction	10° (5°-14°)	5° (−10° to 10°)	5° (−5° to 10°)	10° (6°-21°)
Ulnar abduction	18° (15°-24°)	20° (16° to 29°)	20° (15° to 30°)	25° (17°-34°)
Supination	73° (60°-80°)	63° (56° to 84°)	68° (60° to 70°)	70° (58°-83°)
Pronation	75° (71°-85°)	78° (70° to 84°)	80° (75° to 80°)	75° (65°-80°)
COPM satisfaction	2.4 (1.5-4.0)	4.4 (2.8 to 5.8)*	5.8 (3.8 to 7.5)*	8.5 (7.2-9.0)
COPM performance	4.0 (3.0-5.0)	5.2 (4.0 to 7.2)*	4.0 (3.2 to 6.4)	7.6 (4.9-8.8)
DASH	46 (41-60)	40 (26 to 58)	36 (18 to 42)*	18 (8-25)
PRWE	60 (51-75)	51 (34 to 69)	34 (20 to 50)*	21 (6-24)
VAS activity	8.0 (6.7-8.0)	6.5 (3.5 to 8.0)*	5.0 (4.3 to 8.0)*	5.0 (4.0-8.0)
VAS rest	3.5 (2.0-5.8)	1.5 (1.0 to 4.0)	2.0 (0.0 to 4.5)*	1.0 (0.5-3.0)
Pinch grip, kg	4.5 (3.5-5.0)	4.0 (3.5 to 5.0)	5.5 (3.5 to 6.0)	5.0 (3.0-6.5)
Key pinch grip, kg	5.0 (4.0-8.0)	6.0 (4.0 to 7.5)	6.0 (4.5 to 8.0)	5.5 (3.5-8.0)
Hand grip strength, kg	20 (15-24)	21 (12 to 26)	19 (12 to 30)	16.3

*Note.* Data represented as median (interquartile range). Five-year postoperative data were not included in the statistical analysis. COPM = Canadian Occupational Performance Measure; DASH = Disabilities of the Arm, Shoulder, and Hand; PRWE = Patient-Rated Wrist Evaluation; VAS = Visual Analog Scale.

* Significant improvement (*P* < .05) from preoperative measurements.

** Significant impairment (*P* < .05) from preoperative measurements.

### Secondary Surgery

Secondary surgery was performed 46 times on 16 wrists. The indications varied, but persistent pain was the major cause. In 13 of these 16 wrists, pain was located on the ulnar side of wrist, on the radial side around the first dorsal compartment, or both in combination. We divided the secondary procedures into 3 subgroups (Table 3). The first group included soft tissue surgery, such as median nerve entrapment or wrist radial tendinitis/Morbus de Quervain. In 1 case, persistent volar radial pain was found located over the flexor carpi radialis (FCR) and was successfully treated with FCR tenolysis. The second group included minor skeletal procedures such as intercarpal arthrodesis, ulna shortening, or lunate excision. The third group included revision surgeries. In group 1, there were 11 procedures in 7 patients. In group 2, there were 16 procedures in 9 patients. In 7 cases, excision of the lunate was performed on the indication of persistent ulnar wrist pain. In 3 cases, lunate excision was successful, with reduction of ulnar pain; in 4 cases, lunate excision resulted in persistent pain. In 1 case, excision of a bony fragment was performed on the indication of persistent wrist pain and reduced wrist flexion, with the bony fragment visible on radiographs in the radiocarpal joint. In group 3, 19 procedures were performed on 8 patients. Seven patients were converted to an RCA between 10 and 50 months after the primary RHWA (median, 29 months). All were operated on, with extraction of the implant and arthrodesis. Arthrodesis was performed using a standard arthrodesis locking plate (DePuy Synthes, West Chester, Pennsylvania) and a mix of autologous and homologous bone graft. One patient was converted to a TWA. Of the 7 patients converted to an RCA, 4 healed without complications, while 2 needed several procedures before fusion was achieved. Moreover, 1 patient had an infection after the RCA.

**Table 3.** Reoperations.

Time to reoperation following primary surgery	Group 1 (synovectomy, neurolysis)	Group 2 (lunate excision, carpal arthrodesis)	Group 3 (revision and re-revision surgery)
1 y	3	6	2
1-2 y	6	6	9
2-5 y	2	4	8
Total	11	16	19

*Note.* Data show number of procedures. Ten patients underwent 2 or more reoperations.

## Discussion

We found high rates of secondary surgery following primary surgery using the new RHWA design, and revision surgery is planned for an additional 6 patients. The option of converting the RHWA to a TWA was performed in 1 wrist. We observed a relatively high number of nonunions (3/7) after conversion to an RCA, despite using rigid fixation with a locking plate and bone graft. These failures were reoperated with additional bone grafting, using nonvascularized bone from fresh frozen femoral head allograft. Recent studies report union rates of 95% to 100% following the conversion of TWA to RCA using surgical methods similar to those used in our study—namely, a bridging locking plate in combination with autologous or homologous bone graft.^22,23^ The reasons for our results in achieving union can only be speculated on. However, the loss of bone stock following primary surgery makes the procedure technically demanding.

Most secondary surgeries were performed on the indication of persistent ulnar-sided and/or radial-sided wrist pain. One contributing factor to pain may be the ulnar drift of the carpus, causing increased load on the radial soft tissue structures riding over the implant and secondary radial tendinitis. With ulnar carpal drift and only the scaphoid articulating with the CRF-PEEK-liner, the lunate may impinge on the ulnar head and cause ulnar-sided pain. Performing a PRC at the time of the initial surgery probably could have reduced this issue. The reason for the ulnar translation may likely be related to the sectioning of the radio-scaphoid-capitate (RSC) ligament when preparing the radius for the radial stem. The RSC ligament is known to be an important stabilizer in preventing ulnar translation of the carpus.^24^ The radiographs showed a clear tendency toward ulnar drift of the carpus. To our knowledge, there is no standardized way to assess this phenomenon radiographically. In future studies, this could be further investigated using techniques such as computed tomography–based motion analysis.^25^

Radiolucency was seen around the proximal part of the radial stem in only 1 case. This indicates that the design was successful in promoting osseointegration. However, this conclusion is obstructed by the high rates of revision surgery and only 5 patients completing the 5-year follow-up. Stress-shielding is a mechanism where bone loss is induced by altered load conditions.^18^ This mechanism could be responsible for bone loss around the radial stem in previous wrist arthroplasty designs.^26^ In our study, the distal part of the radial stem has flat proximal surface that rests directly on the cortical bone, potentially reducing the risk of stress-shielding. However, this design requires a transverse cutting of the radial bone, possibly injuring the RSC ligament with potential consequences discussed above.

The CRF-PEEK articulation was used to reduce the risk of wear both on the implant and on articulating bones. However, high rates of bone erosions were found affecting the articulating carpal bones. The cause of this could be 2-fold. First, wear debris from the implant could cause an inflammatory response and secondary osteolysis.^27^ Unfortunately, no tissues samples were taken during revision surgery to assess this microscopically. Second, the CRF-PEEK articulation could cause wear on articulating bones through altered loading conditions, but this is speculative. CRF-PEEK has been evaluated in total hip and total knee arthroplasty designs with mixed results.^28,29^ Karjalainen et al^30^ reported 2 major failures secondary to wear debris using a TWA with metal-on-PEEK articulation, but as far as we know, no hemi-arthroplasty with bone-on-PEEK articulation has been evaluated before.

Only 5 patients with a remaining RWHA implant completed the follow-up. However, all patients completed the 1-year follow-up. The VAS pain scores both at rest and during activity improved significantly at the 2-year follow-up compared with the preoperative values. This is encouraging, as pain is the most important factor in reporting outcomes following wrist surgery.^31^ Although significantly improved, the VAS pain scores during activity were still high in comparison with our experience following TWA,^32^ and the clinical relevance of an improvement in the VAS pain score during activity from 8.0 to 5.0 can be questioned. This is a concern, and the relatively high number of secondary procedures may be related to this finding.

Canadian Occupational Performance Measure satisfaction improved significantly at the 2-year follow-up, compared with preoperative values. The COPM has an advantage over the DASH and PRWE scores in that the patients choose 5 activities that are important for them in their daily life and then grade their performance and their satisfaction with their performance. Issues with both DASH and PRWE in measuring postoperative outcomes following wrist arthroplasty have been discussed in previous studies.^33,34^ The DASH score is designed to measure overall upper extremity disability, and the PRWE involves an evaluation of finger function, which may be a source of misinterpretation, especially in patients with multiple joint disease. In contrast to DASH and PRWE, the COPM score is individualized, which makes it possible to focus on specific wrist problems. The COPM has been shown to be a sensitive tool for capturing clinically important improvement after hand surgery, irrespective of the diagnosis.^35^ Both DASH and PRWE were significantly improved 2 years postoperatively; the DASH score 2 years postoperatively was 36, which is higher than the values previously reported for another hemi-wrist implant.^36^

We found a significant reduction in wrist flexion but no significant change in extension, ulnar/radial deviation, or supination/pronation. Reduced wrist flexion was also found in the previous biomechanical study of the new RHWA design.^14^ One possible explanation for the postoperative reduced flexion could be a dorsal shift in the center of rotation, which was noticed in the biomechanical study. Other studies on wrist hemi-arthroplasties have found wrist ROM to be preserved/increased.^34^ However, the patients in our study had a median flexion of 30°, which is considered sufficient to perform activities of daily living.^37^ Hand grip strength was preserved, and this finding is in line with previous studies on wrist hemi-arthroplasty.^36^

Early in the study process, the implant developers discussed a modulation of the implant involving reduction of the liner radial inclination and replacement of the PEEK material with pyrocarbon, which is a possible solution to the problem of ulnar carpal translation and secondary tendinitis. However, no changes have been made, and the implant is not in use today.

Previous studies concerning hemi-wrist arthroplasties mostly include retrospective series using the radial or carpal component of an existing TWA design. Culp et al^12^ reported on 8 nonrheumatoid patients treated with a hemi-wrist arthroplasty using a radial component with polyethylene articulation in combination with PRC. Seven patients experienced complications—mainly aseptic loosening or reactive synovitis secondary to polyethylene wear. Gaspar et al^11^ retrospectively reviewed 39 patients with a mix of inflammatory and noninflammatory wrist arthritis, who were treated with a hemi-wrist arthroplasty using a radial component with metal articulation in combination with PRC. Complications were reported in 41% (16 of 39) of the wrists after a mean follow-up of 35 months, with revision surgery being performed in 26% (10 of 39). Hemi-wrist arthroplasty using the carpal component of an existing TWA has also been reported, but that study was abandoned due to high failure rate.^38^ In summary, the use of existing TWA components to perform hemi-wrist arthroplasties seems to be accompanied by high complication rates.

Another hemi-wrist arthroplasty with an anatomical design has been evaluated.^39^ Surgery is performed with a ligament-sparing technique in combination with PRC. In a case series of 20 patients, mostly with noninflammatory wrist arthritis, 3 patients underwent revision surgery to TWA or RCA after a mean follow-up of 4.1 years.^36^ Closed manipulation for wrist stiffness was performed in 3 patients. The mean flexion-extension arc was found to improve from 63° to 96° and the mean DASH score from 50.3 to 24.6. These results are favorable compared with the results in our study.

The limitations of this study include the lack of randomization and a control group. The low number of patients who came for the 5-year follow-up may have introduced attrition bias. However, all patients were contacted by phone to assess the number of secondary procedures. The main purpose of this study was to evaluate the clinical outcomes and the possible need for improvement in implant design and instrumentation.

In conclusion, the high frequency of secondary surgery due to persistent pain is a concern, and a modulation of the RHWA design is required before further testing. Further use of the implant in its current design cannot be recommended.
